# 4D PET/CT as a Strategy to Reduce Respiratory Motion Artifacts in FDG-PET/CT

**DOI:** 10.3389/fonc.2014.00205

**Published:** 2014-08-04

**Authors:** Alexander Chi, Nam P. Nguyen

**Affiliations:** ^1^Department of Radiation Oncology, Mary Babb Randolph Cancer Center, West Virginia University, Morgantown, WV, USA; ^2^The International Geriatric Radiotherapy Group, Tucson, AZ, USA

**Keywords:** lung cancer, IGRT, PET, PET/CT, target volume delineation

## Abstract

The improved accuracy in tumor identification with FDG-PET has led to its increased utilization in target volume delineation for radiotherapy treatment planning in the treatment of lung cancer. However, PET/CT has constantly been influenced by respiratory motion-related image degradation, which is especially prominent for small lung tumors in the peri-diaphragmatic regions of the thorax. Here, we describe the current findings on respiratory motion-related image degradation in PET/CT, which may bring uncertainties to target volume delineation for image guided radiotherapy (IGRT) for lung cancer. Furthermore, we describe the evidence suggesting 4D PET/CT to be one strategy to minimize the impact of respiratory motion-related image degradation on tumor target delineation for thoracic IGRT. This, in our opinion, warrants further investigation in future IGRT-based lung cancer trials.

## Introduction

In recent years, ^18^F fluorodeoxyglucose positron emission tomography/computed tomography (FDG-PET/CT) is increasingly used in the delineation of gross tumor for thoracic radiotherapy planning purposes (1, 2). As of now, multiple methods of PET-based tumor volume delineation exist and are being used in clinical practice (3, 4). Although PET/CT may potentially improve tumor delineation, quality and FDG uptake quantification of the PET/CT image are often impaired by respiratory motion. This may lead to decreased accuracy in lung cancer target volume delineation, which decreases the efficacy of thoracic image guided radiotherapy (IGRT), as the uttermost accuracy in tumor localization is desired in IGRT to achieve the most optimal tumor control and normal tissue sparing at the same time. The main cause of this limitation is the difference in the speed of imaging between PET and CT.

In the following sections, the respiratory motion artifacts in PET/CT imaging; and four-dimensional (4D, which accounts for changes in time) PET/CT as a potential strategy to reduce respiratory motion artifacts in the PET/CT imaging of lung cancer will be discussed as this may potentially improve the target volume delineation for thoracic IGRT.

## Respiration Motion Artifacts in FDG-PET/CT

**Figure 1:**
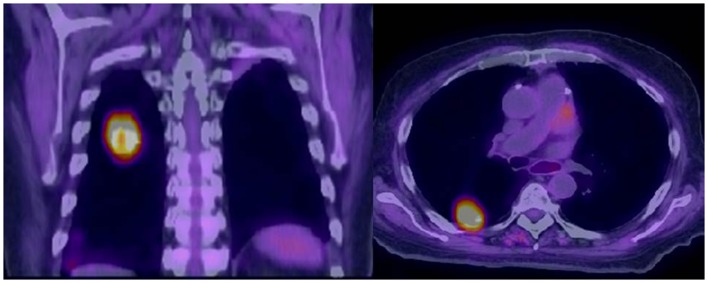
**Misalignment of a recurrent squamous cell carcinoma between free-breathing PET and CT is demonstrated**. The superior portion of the PET avid tumor does not correspond to any anatomically visible tumor on CT.

Computed tomography and positron emission tomography imaging are done at different speeds. While a CT scan can be completed in seconds, the PET is usually done through a sequence of fields of view (FOV) in a matter of several minutes per FOV. As a result, the CT image may capture the lung tumor in a segment of the respiratory cycle only, while the PET image tends to represent an average of the tumor position over several respiratory cycles. This often leads to blurring and/or misrepresentation of the extent of the gross tumor in the registered PET/CT images of the thorax (Figure 1). At the same time, the pattern of FDG uptake intensity within the gross tumor can be changed by respiration, leading to a decrease in the max SUV of the tumor.

Respiratory motion artifacts on PET/CT have been well described in a series of studies (Table 1). In one study, different anatomical points between the apices and the diaphragmatic domes of the lungs on PET/CTs were identified and matched (5). For each patient, the PET was registered to CTs obtained during maximum inspiration, maximum expiration, free breathing, and normal expiration. Prominent mismatch between FDG-PET and CT was found during maximum inspiration and maximum expiration, and the best PET/CT co-registration occurred when the CT was obtained during normal expiration (5). Mismatch between PET and CT was most prominent at the diaphragmatic dome. On average, it was 0.4 mm with the CT taken during normal expiration, but 44.4 mm with the CT taken during maximum inspiration (*p* = 0.001). This has a direct influence on image registration for tumor in PET/CT.

**Table 1:** **Motion artifacts observed in FDG-PET/CT**.

	Artifacts observed
Goerres et al. (5)	Mismatch of the diaphragmatic dome between PET and CT was 44.4 ± 25.5 mm between free-breathing PET and CT obtained during maximum inspiration. It ranged between 8.7 and 82.9 mm
Goerres et al. (6)	Mismatch of NSCLC lesions was most prominent at the lung periphery:
	Breathing: 6.5 ± 3.6 mm (3.4–14.7 mm)
	Breath hold: 6 ± 2.9 mm (0.5–11.4 mm)
	And the lung base:
	Breathing: 8.2 ± 1 mm (7.2–9.8 mm)
	Breath hold: 6.2 ± 2.6 mm (2.9–11.3 mm)
Cohade et al. (7)	Misregistration of free-breathing PET and CT was 7.55 ± 4.73 mm
	Lower lungs: 10.2 ± 6.55 mm
	Upper lungs: 6.67 ± 4.28 mm
	Left lung: 8.33 ± 5.05 mm
	Right lung: 6.25 ± 3.92 mm
Osman et al. (8)	Six patients with liver metastases at the liver dome mislocalized to the right lower lobe of the lung on free-breathing PET
Goerres et al. (9)	PET attenuation by CT taken during maximum inspiration led to a decrease in FDG concentration in lung tumors by 42 ± 12% when compared to that attenuated by CT taken during maximum expiration
Erdi et al. (10)	Tumor displacement of 6.4–24.7 mm, and tumor maximum SUV reduction of 6–24% were observed between maximum inspiration and expiration
Nehmeh et al. (11)	4D PET can led to a 28% reduction in tumor volume and 56.5% increase in tumor maximum SUV in a patient when compared to free-breathing PET/CT
Liu et al. (12)	Diaphragmatic motion of 11 mm can cause maximum SUV underestimation of 28% and tumor volume overestimation of 130% on average for 1 cm lung lesions

In another study, the accuracy of PET and CT registration for non-small cell lung cancer was investigated with CT obtained during shallow breathing or normal expiration (6). The most prominent incongruence of gross tumor on CT and on PET was observed at the periphery and the base of the lungs (0.5–14.7, 2.9–11.3 mm, respectively). Much less incongruence was observed in the central and apical regions of the lungs (1.7–5.4, 0.7–5.9 mm, respectively) (*p* < 0.0001). In this study, normal expiration appears to be better than shallow breathing for PET/CT matching (*p* = 0.024).

Errors in spatial registration of lung lesions on PET/CT were also observed in free-breathing (FB) patients by Cohade et al. (7). In their study, the distance between the center of lung lesions defined on PET and CT was 7.55 mm on average, which tended to be more pronounced in lower lobe tumors. As shown in these studies, errors in PET/CT registration may become significant in the peri-diaphragmatic region, which can lead to mislocalization of a tumor into an adjacent organ in extreme cases (8).

In addition to image registration mismatch, respiratory motion can also lead to a decrease in measured FDG concentration in lung tumors, which may be further confounded by other factors, such as the body size, blood glucose concentration, uptake time allowed, interscanner variability, and image reconstruction parameters (9, 13). This is well described by Erdi et al. when PET was registered with respiration-corrected CT over 10 phases (10). Between maximum inspiration and expiration, lesion displacement of 6.4–24.7 mm was observed on 4D CT, which correlated with decrease in tumor maximum SUV of 6–24% between end inspiration and end expiration. The reduction of FDG uptake intensity observed is due to a redistribution of FDG activity concentration over the range of respiratory motion, which leads to a drop in maximum activity concentration within the tumor (11). As FDG uptake distribution within a tumor is altered, the shape of the gross tumor may also be altered, leading to an increase in tumor size on PET.

The respiratory motion induced change in FDG uptake intensity is further demonstrated in an analysis of routine PET/CT studies in correlation with over 1000 respiratory traces that is validated in a phantom study (12). In this study, a mean maximum SUV underestimation of 28% and mean lesion volume overestimation of 130% in PET/CT images of 1 cm lesions were observed when respiratory motion at the diaphragm is 11 mm. The underestimation of FDG intensity appears to be proportional to respiratory motion amplitude.

In the same study, PET/CT mismatch-related FDG intensity overestimation for lower lobe lung lesions located close to the dome of the liver was also reported. The fluctuation in activity concentration within the tumor may potentially decrease the quality and capability of tumor imaging, which may affect the appropriate delineation of the gross tumor volume (GTV) during radiotherapy treatment planning.

Various strategies have been proposed to reduce respiratory motion artifacts in PET/CT. Among them, PET registered with a respiratory motion averaged CT has been proposed by Chi et al. (14). Motion averaged CT is conducted following routine PET/CT in 229 lung cancer patients in this study. Image alignment and tumor quantification were analyzed in 216 of these patients. Image misalignment was observed in 68% of the 216 routine PET/CTs, which was completely removed (86.21%) or reduced (13.79%) with PET and motion averaged CT registration. Among 120 PET/CTs in which the GTV can be delineated, alignment correction with motion averaged CT was associated with changes in the maximum SUV and GTV of up to 73 and 1950% between PET registered with regular CT and PET registered with motion averaged CT. The largest variation was observed in small lesions <50 cm^3^ in the vicinity of the diaphragm. Thus, demonstrating the potential for PET attenuated/registered with motion averaged CT to enhance tumor quantification on PET, which is also suggested in a study of 80 patients with NSCLC by Cheng et al. (15).

An alternative approach to reducing respiratory motion-related PET/CT image degradation is PET/CT acquisition through repeated imaging during breath holding (16, 17). However, this method may be limited by patient compliance if used for tumor volume delineation in patients with NSCLC due to their poor pulmonary function.

On the other hand, such image degradation can be more easily reduced by the generation of a respiratory motion corrected or 4D PET/CT during which the PET data are acquired in synchronization with respiratory motion, which can also minimize the poor image quality of PET/motion averaged CT in the vicinity of soft tissue, such as the diaphragm and the chest wall (11, 18, 19). 4D PET/CT is done through tracking an external tracer block that is placed on a patient’s abdomen at the time of image acquisition as observed in 4D CT. Through 4D PET/CT imaging, prominent increase in FDG intensity of the tumor and reduction of tumor size due to decreased smearing effect have been observed (11, 20–22). This may potentially increase the accuracy of GTV delineation on FDG-PET/CT and decrease the amount of normal tissue included in the GTV. As a result, increased accuracy for tumor localization and radiation dose escalation with IGRT for NSCLC may be possible.

## 4D FDG-PET in Target Volume Delineation for IGRT

A very high degree of accuracy is required in the target volume delineation to optimize accurate tumor localization and maximal margin reduction in the delivery of image guided, intensity-modulated radiotherapy (IMRT) for lung cancer. Respiratory motion-related image degradation observed in FB PET/CT may have an impact on the accuracy of target volume delineation for sophisticated treatments, such as IGRT. In the planning of stereotactic ablative radiotherapy (SABR) for early-stage NSCLC, FB PET-target volumes often do not entirely encompass tumor motion, as FB-PET tumor volumes are often smaller than ITVs generated from the maximum intensity projections (MIP) obtained from 4D CT (23). Thus, target volumes delineated on FB-PET may increase the risk of geometric misses. This is especially problematic for image guided SABR, because of the high reliance on accurate tumor localization and the potential consequences of severe toxicity due to such misses. However, this problem may be corrected by 4D PET/CT imaging (22).

With the utilization of 4D FDG-PET or PET/CT, the most PET active subvolumes within the tumor may be more consistently identified for dose painting (24). In addition, missing disease undetectable on FB PET due to image degradation may be prevented with 4D PET/CT in the planning of thoracic IGRT in order to achieve the maximal TCP (25). 4D PET MIP for ITV delineation was described in detail by Lam et al. (18). ITV generated from 4D PET MIP was found to better correlate with that generated from 4D CT MIP than FB-PET-based ITV in this study. Furthermore, better definition of the extent of tumor in the vicinity of mobile structures, such as the diaphragm, the heart, and the chest wall with 4D PET was demonstrated, which can potentially improve the accuracy of tumor target definition and the sparing of normal tissue that is of similar density to the tumor.

Similarly, 4D PET-based ITV generation may improve the identification of tumor motion in the hilar and mediastinal regions over CT alone. Respiratory motion has been known to affect mediastinal and hilar lymph nodes (26). However, the similar tissue density between nodal disease and surrounding normal tissue in the mediastinal region may pose a challenge to ITV definition based on 4D CT alone. In a comparison of ITV generation based on 3D PET and 4D PET, a 1.3 cm expansion was required for ITV_3D_ to include 91% of the nodal disease (27). Further analysis of the ITV_3D_ with 4D PET demonstrated the inclusion of 45 ± 34 cm^3^ non-PET-avid tissues in the 3D PET volume. Therefore, 4D PET-based nodal ITV may further improve the accuracy of nodal disease definition, leading to the sparing of additional normal tissue adjacent to regional nodal disease. This may further improve the therapeutic ratio, especially with IGRT in the treatment of lung cancer.

The current evidence, described above, suggests that target volume delineation based on 4D PET/CT information may be the best approach currently available for the delineation of tumor volumes for lung cancer. It warrants further investigation in future prospective studies, especially in the setting of dose escalation. In our opinion, its use in the clinical setting whenever possible is strongly encouraged, as it may improve patient treatment outcome in the setting of IGRT for lung cancer.

## Conflict of Interest Statement

The authors declare that the research was conducted in the absence of any commercial or financial relationships that could be construed as a potential conflict of interest.
